# Translational Potential of Epigenetic-Based Markers on Fine-Needle Aspiration Thyroid Specimens

**DOI:** 10.3389/fmed.2021.640460

**Published:** 2021-03-23

**Authors:** Sule Canberk, Ana Rita Lima, Mafalda Pinto, Valdemar Máximo

**Affiliations:** ^1^Instituto de Investigação e Inovação em Saúde (i3S), University of Porto, Porto, Portugal; ^2^Cancer Signalling and Metabolism Group, Institute of Molecular Pathology and Immunology of the University of Porto (Ipatimup), Porto, Portugal; ^3^Abel Salazar Institute of Biomedical Sciences (ICBAS), University of Porto, Porto, Portugal; ^4^Faculty of Medicine of the University of Porto (FMUP), Alameda Prof. Hernâni Monteiro, Porto, Portugal; ^5^Department of Pathology, Faculty of Medicine of the University of Porto (FMUP), Alameda Prof. Hernâni Monteiro, Porto, Portugal

**Keywords:** epigenetics, epigenomics, thyroid tumor, thyroid cancer, thyroid fine needle aspiration, epigenetic markers of thyroid

## Abstract

The awareness of epigenetic alterations leading to neoplasia attracted the attention of researchers toward its potential use in the management of cancer, from diagnosis to prognosis and prediction of response to therapies. Our group has focused its attention on the epigenomics of thyroid neoplasms. Although most of the epigenetic studies have been applied on histological samples, the fact is that cytology, through fine-needle aspiration, is a primary diagnostic method for many pathologies, of which thyroid nodules are one of the most paradigmatic examples. This has led to an increasing literature report of epigenetic studies using these biological samples over the past decade. In this review, our group aimed to document recent research of epigenetic alterations and its associated assessment techniques, based on cytology material. Our review covers the main epigenetic categories—DNA methylation, histone modification, and RNA-silencing—whose evidence in thyroid cytology samples may represent solid soil for future prospectively designed studies aiming at validating patterns of epigenetic alterations and their potential use in the clinical management of thyroid neoplasms.

## Introduction

Epigenetic alterations are potential candidates for the identification of specific markers for cancer detection, diagnosis, and prognosis (1). The utility of epigenetic markers in thyroid cytology should be scoped under the diagnostic, prognostic, and predictive translational extensions, aiming for a more personalized management of patients with a thyroid nodule. For thyroid cytologists, two key questions need to be answered in clinical practice: (1) Is this a case within the group of follicular-patterned lesions? (2) Does this malignant case hold an aggressive morphology? The former question assumes that benign, precursor, or borderline (low-grade malignancies) categories are generally encompassed in a gray zone group of lesions. This is the category that downgrades the diagnostic power of thyroid cytology. To make this first question concrete, if we consider patients with an increased thyroid suppression hormone (TSH) and with a cytology suspicious for follicular or Hürthle cell neoplasm, they should undergo lobectomy or total thyroidectomy, unless a molecular testing would predict a low risk of malignancy. When a surgical decision is made, one needs to consider risk factors for Thyroid carcinoma (TC), clinical and imaging considerations, and patients' informed consent about benefits and risks of diagnostic procedures, including surgery. Surgical complications increase when performing total thyroidectomy compared to lobectomy (2, 3). Prognosis and the need to anticipate risk of recurrence and metastatic disease are clinical challenges that fall under the scope of the second question. Despite the diagnostic power of fine-needle aspiration (FNA) as a gold-standard technique for the presurgical diagnosis of thyroid nodules, the diagnosis of indeterminate categories, especially the lesions falling under atypia of undetermined significance/follicular lesion of undetermined significance and suspicious for follicular neoplasm/follicular neoplasm categories, still remains a challenge in cytopathology practice. Indeed, ~30% of the cases lack the morphological features to provide a definitive classification and are diagnosed as “indeterminate.” With the purpose of reducing unnecessary surgery and predicting malignancy, many panels based on genetic alterations were developed in the past decades (4). These commercially available tests were built on the concept of either rule out or rule in malignancy, based on high-throughput technologies—a targeted next-generation sequencing (NGS) of DNA and RNA. Despite the improvement of diagnosis, ~30% of TC cases are in a genetic “dark zone,” with no well-established driver mutations.

In the past two decades, TC research has started to enlighten the contribution of epigenetic changes to the deregulation of gene transcription and its link with oncogenic pathway activation. The available evidences point to the fact that, beyond genetic factors, the differentiation and proliferation traits of TC cells are strongly influenced by epigenetic alterations. As an example, *PTEN* promoter presents with hypermethylation in ~50% of papillary thyroid carcinomas (PTCs) and nearly 100% of follicular thyroid carcinomas (FTCs) and follicular adenomas (FAs), suggesting that it may be involved in thyroid tumorigenesis (5). Activating mutations of *BRAF* in PTCs were linked to altered methylation of other genes, including *TIMP3, SLC5A8, DAPK*, and *RARb2* (6), which were associated with an aggressive behavior in thyroid neoplasms (7). Approximately 30% of benign and malignant thyroid tumors, including anaplastic thyroid carcinoma (ATC), present promoter methylation involving the *RAS* association family 1A (*RASSF1A*) tumor-suppressor gene (8–10), known to have a role in regulating several key cell processes, suggesting that this change may occur early in the tumorigenesis.

In this review, our group aimed to provide a brief overview of the epigenetic alterations and their translational potential on thyroid cytology specimens.

## Epigenetic Mechanisms Contributing to Thyroid Tumourogenesis

The term “epigenetic” was eloquently coined by Waddington (11) in 1942 as describing heritable changes inside the genome leading to an altered gene expression pattern without affecting the main core of DNA. “DNA methylation,” “histone modification,” and “RNA silencing” are considered as main mechanisms of epigenetic regulation of gene expression through the modulation of chromatin structure. In particular, DNA methylation and histone modification are not independent mechanisms; they both act in an orchestrated fashion to regulate chromatin states by using DNA methyltransferases (DNMTs) and a large number of histone-modifying enzymes. Chromatin structure has two different states, “heterochromatin” (a closed chromatin), which is associated with transcriptional repression, and “euchromatin” (an open chromatin) favorable to transcription (12). Each of these effector mechanisms involves enzymes that transfer the modification (“writers”), enzymes that modify or revert a modification (“erasers”), and enzymes that mediate the interactions of proteins or protein complexes with the modification (“readers”), contributing directly or indirectly, through the creation or elimination of protein- or protein complex–binding sites regulating gene expression, to the ectopic transcription of several genes, including oncogenes or proto-oncogenes, or the suppression of tumor suppressor gene transcription. Cancer presents with frequent alterations to the epigenome, including mutations in genes controlling the epigenetic players, silencing several tumor suppressor genes whose roles are implicated in almost all cancer-relevant signaling pathways, such as apoptosis, cell proliferation, cell migration, and DNA repair (13, 14). It has also been well-documented that epigenetic alterations play a significant role in the differentiation and proliferation properties of thyroid cancer (11, 12). Of those, DNA methylation is the most widely studied mechanism, which is responsible for adding a methyl group to the 5′ position of cytosine, predominantly in the context of CpG dinucleotides. Aberrant DNA methylation is the prevalent epigenetic dysregulation in cancer, consisting of both losses (DNA hypomethylation) and gains (DNA hypermethylation) of 5-methyl-cytosine within the CpG dinucleotides. As DNA methylation, histone modification patterns are also dysregulated in cancer. Some histone modifications are associated with gene activation (i.e., trimethylation on histone H3, lysine 4 [H3K4me3]), and others are associated with gene repression (i.e., H3K9me3) (15). Histone modifications are posttranslational modifications, including acetylation, methylation, phosphorylation, ubiquitylation, and glycosylation, among others (15, 16). Histone modifications are the result of the balance between different groups of enzymes, some with antagonist activity. Acetyl groups are added to lysine residues in histone tails by enzymes called histone acetyltransferases causing chromatin decondensation, generally leading to gene activation, and are removed by histone deacetylases (HDACs) (15). Methyl groups are added to lysine and arginine residues by histone methyltransferases, which promote or inhibit transcription, depending on the residue alteration. As an example, methylation of lysine 9 of histone 3 leads to repression, whereas methylation of lysine 4 of the same histone results in gene activation. Methyl groups are removed by histone demethylases (15, 17).

In the last decade, non-coding RNAs (ncRNAs) have been linked to the development and progression of cancer and have been proposed as markers for diagnosis, including of TC (18–24). They can be classified as small interfering RNAs, microRNAs (miRNAs), piwi-associated RNAs, long ncRNAs (lncRNAs), and enhancer RNAs. Of those, the most well-studied ncRNAs are miRNAs, which are known to have either oncogenic or tumor-suppressive roles (25, 26). Interestingly, mutations in *DICER1*, a member of the RNase III family with a pivotal role in the maturation of miRNAs, have already been described in different human tumors, including TC (27); miRNAs are small single-stranded ncRNAs of ~22 nucleotides transcribed from endogenous DNA and later processed to mature miRNAs, which target and bind to transcripts interfering with protein translation or causing messenger RNA degradation, with an impact on protein production (28).

## Translational Application of Epigenetic Alterations in TC

### Contribution of Epigenetic to Cytology Diagnostic Challenges

FNA is a standard method for diagnosis of thyroid nodules, but it holds some limitations. Evidence exists supporting the hypothesis that epigenomic profiling could identify markers to differentiate benign from malignant thyroid neoplasms.

DNA methylation is an epigenetic mechanism normally tested in the diagnosis of thyroid nodules. Keelawat et al. (29) assessed the global hypermethylation status in a series of 15 FAs, 18 FTCs, and 17 PTCs. Although no statistically significant difference was found in methylation levels between FAs, FTCs, and PTCs (*P* = 0.44), as assessed by a combined bisulfite restriction analysis polymerase chain reaction (PCR) protocol, the immunohistochemical staining 5-methylcytidine score was significantly higher in tumors in comparison with normal tissue counterparts, for FAs (*P* < 0.001), FTCs (*P* = 0.04), and PTCs (*P* = 0.02). Interestingly, PTCs showed the highest expression among all other tumors, which was significantly different from FTCs (*P* = 0.015), but not FAs (*P* = 0.09) (29). Several studies have addressed the methylation pattern in thyroid tumors and have demonstrated variable results. DNA hypomethylation is not involved in the development of well-differentiated thyroid cancer; nor is it involved in progression from benign (adenoma) to malignant disease (carcinoma). In line with other tumors, such as renal cell carcinoma, TC is associated with global hypermethylation, contributing to the silencing of tumor suppressor genes (29). Stephen et al. (30) analyzed 21 genes for the presence of methylation in FNA and corresponding matched postsurgical fresh thyroid tissue from two cases, using quantitative methylation-specific PCR, and have identified 6 genes (*NIS RASSF1, TSHR, SERPINB5, SLC26A4, TPO*), which represented a concordant presence of methylation results. This study pointed to the usefulness of methylation markers where FNA shows limitations by the histopathologic nature of lesions.

In 2008, Nikiforova et al. (24) analyzed miRs expression profiles in FNA material. They found a distinctive expression pattern associated with FTCs by real-time PCR using the TaqManTM MicroRNA panel: miR-155,−187,−221,−222, and−224 were found to be highly overexpressed in conventional FTCs, whereas miR-183,−187,−197,−221,−222, and−339 were overexpressed in the oncocytic variants of FTCs, in 60 surgically removed thyroid neoplastic and non-neoplastic samples and in 62 FNA samples. Mazeh et al. (31) analyzed the miRNA expression profiles of 79 malignant and 195 benign thyroid nodules by NGS. The authors selected a 19 miRNAs-based diagnostic panel whose expression was statistically different between benign and malignant samples. This panel was validated in 35 thyroid nodules (22 malignant and 13 benign nodules), which were previously reported as indeterminate in cytology, presenting sensitivity, specificity, negative predictive value (NPV), positive predictive value, and overall accuracy scores of 91, 100, 87, 100, and 94%, respectively. Noteworthy, the overall accuracy of this panel is potentially higher than the commercially available genetic tests. There are also miRNA-based commercially available tests such as ThyraMIR®, a miRNA gene expression classifier based on 10 miRNAs detected by PCR. Similarly, the RosettaGX Reveal™ assays 24 miRNAs and is also designed to stratify indeterminate thyroid nodules as benign or suspicious for malignancy by using a single FNA stained smear (32). Although quality assurance review of the commercially available tests cannot be underemphasized, only few literature reports assessed the performance of such tests (33). More recently, in a validation study of a new miRNA-based thyroid molecular classifier test (mir-THYpe) based on the expression of 96 miRNA candidates using FNA smear slides, a performance comparison with other five molecular classifiers was done (34). All tests—genomic and miRNA-based—were predicted to perform with an NPV >90% assuming the cancer prevalence used by the genomic Afirma GSC study (23.7%) (34, 35). However, with the cancer prevalence used by the ThyroSeq v3 study (52.6%), only mir-THYpe, ThyroSeq v3, and ThyroidPrint tests were predicted to have an NPV higher than 90% (34, 36). With mir-THYpe, out of the 76 cancer and 97 benign lesions, 70 and 83, respectively, were correctly classified (34). Expression deregulation of lncRNAs has an important role in carcinogenesis. Besides their involvement in genomic imprinting, inactivation of chromosome X, maintenance of pluripotency, and the formation of different organs via changes in chromatin, transcription, and translation, they are also known to act as tumor suppressor genes or oncogenes. Recently, Possieri et al. (37) analyzed six cancer-associated lncRNAs (MALAT1, NEAT1, HOTAIR, H19, PVT1, MEG3) in 135 FNA samples, with MALAT1, PVT1, and HOTAIR showing a significant differentiation capability between malignant and benign nodules (*P* < 0.0001).

### Contribution of Epigenetic to TC Patient Management

The utility of thyroid cytological samples' epigenetic signatures for the management of TC can be seen under the lens of an improvement of prognosis and disease risk stratification, or under a more farfetched objective—targeting epigenetic changes as a mechanism of disease to modulate cancer hallmarks with a treatment intent. The former bases its usefulness on the assumption that specific epigenetic marks detected on a cytology sample could guide the surgical approach and the decision to implement adjuvant treatment with radioactive iodine (RAI), including dosages, and TSH suppression. The latter assumes that epigenetic changes can be reverted by means of a pharmacological compound with the possibility of reverting radioiodine refractoriness, potentially prolonging disease-free survival (DFS).

#### Disease Prognosis and Risk Stratification

Under the context of cytology specimens, epigenetic alterations should be able to support a presurgical prognostic assessment to be applied in clinical practice, as validated in the form of a molecular signature. Such clinical application would ideally cover Bethesda categories III–VI, where not only the malignancy risk is increasingly higher, but also the need for an assessment of estimated risk of recurrence is needed. One of the main treatment objectives of TC is to minimize the risk of recurrence and metastatic dissemination, and therefore adequate surgery is the most important treatment influencing prognosis (38).

Within the prognosis and risk stratification fields, the most promising epigenetic markers that can be detected in FNA are miRNAs. Yip et al. (23) showed that miR-146b and miR-222 upregulation and miR-34b and miR-130b downregulation were associated with aggressive behavior of PTCs. In their study, miR-146b showed a strong association with aggressive PTCs in *BRAF*-positive tumors, and *MET* was identified as a potential target for the downregulated miR-34b and miR-130b, with a significantly higher *MET* expression in aggressive PTCs. Chou et al. (39) have also shown that high levels of miR-221, miR-222, and miR-146b expression correlated with extrathyroidal invasion in PTCs. In addition, Dettmer et al. (20) have reported an upregulation of miR-183-3p in poorly differentiated thyroid carcinoma (PDTC) in comparison with well-differentiated PTCs or follicular variants of PTC, which was also associated with decreased patient survival. In this study, a significant association with tumor relapse and tumor-specific death was found for miR-23b and miR-150, respectively, in PDTC. Petric et al. (18) analyzed a series of Hürthle cell carcinoma (HCCs) and have found that miR-138 and miR-768-3p were both downregulated in HCC in tumors with metastases vs. those without metastases (*P* = 0.030 and *P* = 0.048, respectively). In addition, they showed that miR-183, miR-221, and miR885-5p were significantly downregulated in HCC with metastases (*P* = 0.027, *P* = 0.019, and *P* = 0.024, respectively).

Regarding methylation markers indicative of invasive disease, Stephen et al. (30) have found that *DAPK1* and *ESR1* methylation were significantly associated with extrathyroidal extension (*P* = 0.014 and *P*= 0.036, respectively) in FTC and HCC cases. In the same study, methylation of *DAPK1* and *ESR1* was significantly associated with late-stage disease (*P* = 0.034 and *P* = 0.035, respectively). Various studies have shown the potential of *RASSF1A* hypermethylation as a biomarker of aggressive tumors (8, 40), both in PTCs and FTCs, including the meta-analysis by Niu et al. (41), where the *RASSF1A* promoter methylation was found to be associated with poor DFS. These data support *RASSF1A* methylation as a putative epigenetic prognostic biomarker. Noteworthy, Mancikova et al. (42) had previously proposed that etoposide-induced 2.4 (*EI24*) and Wilms tumor 1 (*WT1*) could be candidate prognostic markers related to recurrence-free survival both in PTCs and FTCs. On the same study, kallikrein 10 gene (*KLK10*) was hypomethylated and overexpressed in *BRAF*-mutated tumors, which was recently supported by the evidence by Buj et al. (43), showing that the overall *KLK* family is altered in PTCs, with specific epigenetic marks strongly associated with *BRAF*^V600E^ or *RAS* mutations. In this study and based on a proposed *KLK* algorithm, a new PTC subtype emerged showing favorable prognosis. More recently, Klein Hesselink et al. (44) reported an increasing Alu hypomethylation in distant metastatic DTC, PDTC, and ATC, as compared to low-risk DTC and pediatric PTCs, which did not show the same hypomethylation pattern. According to the authors, this could involve a global pattern of hypomethylation in a subset of TC with advanced disease and cell dedifferentiation.

Although with a prerequired prospective validation, the assessment of these markers could further help characterize the invasive or aggressive nature of the primary tumor and support the decision of adopting a more intensive surgical approach and further guide the need for RAI remnant ablation, adjuvant RAI treatment and dosage, and the level of TSH suppression.

#### Epigenetic Changes as Therapeutic Target in TC

Russo et al. (45) have elegantly summarized the main epigenetic strategies potentially applied to TC treatment: (1) based on the redifferentiation of tumors while resensitizing them to radioiodine therapy and (2) through the epigenetic activation of tumor suppressors affecting cell proliferation, growth, and invasion. In particular, evidence showing hypermethylation-based silencing of key iodine-handling genes, such as *NIS* and *TSHR*, has been reported (46–49), including in proteins that seem to be involved in the transport of iodine in the apical membrane of thyrocytes (6, 50). The thyroid master regulator *TTF1* gene promoter was also found to be hypermethylated and was a target of histone modifications in PDTC (51). Interestingly, the use of DNMT (46, 48, 52) and HDAC inhibitors *in vitro* has reverted the expression of these genes (53–60).

The majority of the compounds that have shown to reverse epigenetic alterations inhibit either DNMT or HDAC, and some are already being used in clinical practice in hematologic malignancies. Most of those drugs alter acetylation and DNA methylation, thereby having an effect on cancer differentiation and proliferation. Many phases 1–2 trials are being conducted to assess the effect of DNMT and HDAC inhibitors in patients with TC and other solid tumors. Finally, in what treatment application is concern, mi-RNAs are still far from being a druggable target, and most evidence of its antitumoral effect in TC is based on *in vitro* studies (19).

## Epigenetic Techniques for FNA Materials

For DNA methylation detection, cytology smears or cell blocks can be used for bisulfite sequencing, methylation-specific PCR, real-time PCR, immunostaining, and NGS. Advantages and disadvantages of these techniques are outlined in Table 1.

**Table 1:** Advantages and limitations of the different epigenetic detection techniques.

**Epigenetic alteration**	**Technique**	**Advantages**	**Limitations**
DNA methylation	Bisulfite sequencing (BS) (61)	Highly quantitativeSingle-site resolution	Dedicated equipment
	Methylation-specific PCR (MSP) (62)	InexpensiveEasy to perform	Qualitative/semiquantitative No single-site resolution
	Real-time qPCR (63)	QuantitativeEquipment easily accessible	Low precision No single-site resolution
	Immunostaining (64, 65)	Allows the visualization of methylation pattern simultaneously in normal adjacent tissue	Need for specific antibodies Lower sensitivity
	NGS (66–69)	AccurateHigh throughputFast	Very expensive Dedicated equipment Need for bioinformatics experts
Chromatin modification	DNase sensitivity (70, 71)	Inexpensive	Limited to known regions of DNA
	ChiP (72)	Evidence of endogenous interaction at a specific geneHigh sensitivityDo not require expensive instrumentationQuick techniqueAllows the characterization of DNA–protein interactions *(*ChIP-Seq)	Requires a specific and validated antibody; crosslinking may induce artifacts Only known modifications can be studied Limited by the availability, specificity, and performance of antibodies Lack high-throughput capabilities High cost for large-scale studies
	Mass spectrometry (73)	Identify multiple modifications in single peptidesAllows simultaneously monitoring of multiple PTMsAccurate relative quantification of global changes of histone PTMsAllow discovery of previously unknown modification patternsCan be used in a high-throughput manner	Does not require specific antibodies Laborious Limited in high sensitivity High expertise is required for data analysis Validation of the MS results with antibody-based techniques is still recommended, as it is more widely accepted strategy
miRNA detection	Real-time qPCR (74, 75)	Needs only a small amount of starting materialQuantitativeEquipment easily accessible	Low precision Cost
	Arrays (76)	High throughput	Requires miRNA library
	NGS (77–79)	Allows expression and sequencing at the same timeHigh throughput	Requires high-quality RNA Requires miRNA library Very expensive Dedicated equipment Need for bioinformatics experts

**Figure 1:**
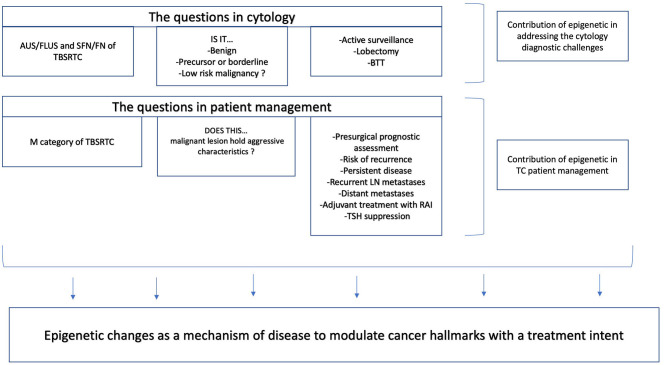
Translational potential of epigenetic markers based on fine-needle aspiration (FNA) thyroid specimens.

Although all these techniques can be applied on cytology material, the presence of “contaminant cells” can be a practical issue. Studies have shown that 17% of thyroid nodules miss malignant- or benign-specific DNA methylation changes. The absence of epigenetic signatures is frequently linked to lymphocytic thyroiditis. In fact, a group of thyroid nodules with thyroiditis had a DNA methylation pattern very similar to lymph nodes (Figure 1). In addition, 62% of adjacent thyroid tissues and 40% (2 of 5) of malignant tissues with 75% or more of adjacent thyroid tissues were undiagnosable according to Diagnostic DNA Methylation Signature. These data suggest that the presence of cells other than follicular epithelial cells in a specimen may lead to the absence of epigenetic signatures. Such contamination is frequently found in FNA biopsies of thyroid nodules and may include white blood cells, skeletal muscle, blood vessels, or adjacent normal tissue (80).

## Conclusions

Despite the improvement of diagnosis through these molecular advances, even the most recent molecular tests present a wide confidence interval for cancer probability, resulting in a lack of established marker(s) that either provide a clear-cut diagnosis or accurately predict the prognosis. Meanwhile, advances in the epigenetic field supporting the differentiation between benign, borderline, and malignant lesions, as well as their role as prognostic factors in malignant neoplasms of various organ systems, such as breast, prostate, lung, or liver, are increasingly acknowledged. Recently, thyroid also started to be studied by various epigenetic techniques. The application of epigenetic-based methods on tissue material of thyroid and various organs has been targeted by researchers in the past two decades, leading to a growing need to establish its applicability to FNA material. Following what has been reported so far, this review amassed the potential usage and adoption of epigenetic-based techniques on thyroid FNA material aimed to better understand the oncobiology of thyroid neoplasia.

## Author Contributions

SC and AL: conceptualization. SC, AL, and MP: writing – original draft. SC, AL, MP, and VM: writing – review & editing. VM: supervising. All authors contributed to the article and approved the submitted version.

## Conflict of Interest

The authors declare that the research was conducted in the absence of any commercial or financial relationships that could be construed as a potential conflict of interest.

## References

[1] Jones PA Baylin SB. The fundamental role of epigenetic events in cancer. Nat Rev Genet. (2002) 3:415–28. 10.1038/nrg816 12042769

[2] Haugen BR. 2015 American thyroid association management guidelines for adult patients with thyroid nodules and differentiated thyroid cancer: what is new and what has changed? Cancer. (2017) 123:372–81. 10.1002/cncr.30360 27741354

[3] Hauch A Al-Qurayshi Z Randolph G Kandil E. Total thyroidectomy is associated with increased risk of complications for low- and high-volume surgeons. Ann Surg Oncol. (2014) 21:3844–52. 10.1245/s10434-014-3846-8 24943236

[4] Muzza M Colombo C Pogliaghi G Karapanou O Fugazzola L. Molecular markers for the classification of cytologically indeterminate thyroid nodules. J Endocrinol Invest. (2020) 43:703–16. 10.1007/s40618-019-01164-w 31853887

[5] PTEN promoter methylation in sporadic thyroid carcinomas. Thyroid. (2006) 16:17–23. 10.1089/thy.2006.16.17 16487009

[6] Hu S Liu D Tufano RP Carson KA Rosenbaum E Cohen Y. Association of aberrant methylation of tumor suppressor genes with tumor aggressiveness and BRAF mutation in papillary thyroid cancer. Int J Cancer. (2006) 119:2322–9. 10.1002/ijc.22110 16858683

[7] Xing M. Gene methylation in thyroid tumorigenesis. Endocrinology. (2007) 148:948–53. 10.1210/en.2006-0927 16946009

[8] Schagdarsurengin U Gimm O Hoang-Vu C Dralle H Pfeifer GP Dammann R. Frequent epigenetic silencing of the CpG island promoter of RASSF1A in thyroid carcinoma. Cancer Res. (2002) 62:3698–701. 12097277

[9] Nakamura N Carney JA Jin L Kajita S Pallares J Zhang H. RASSF1A and NORE1A methylation and BRAFV600E mutations in thyroid tumors. Lab Invest. (2005) 85:1065–75. 10.1038/labinvest.3700306 15980887

[10] Xing M Cohen Y Mambo E Tallini G Udelsman R Ladenson PW. Early occurrence of RASSF1A hypermethylation and its mutual exclusion with BRAF mutation in thyroid tumorigenesis. Cancer Res. (2004) 64:1664–8. 10.1158/0008-5472.CAN-03-3242 14996725

[11] Waddington CH. The epigenotype. 1942. Int J Epidemiol. (2012) 41:10–3. 10.1093/ije/dyr184 22186258

[12] Biswas S Rao CM. Epigenetic tools (The Writers, The Readers and The Erasers) and their implications in cancer therapy. Eur J Pharmacol. (2018) 837:8–24. 10.1016/j.ejphar.2018.08.021 30125562

[13] Biswas S Rao CM. Epigenetics in cancer: fundamentals and beyond. Pharmacol Ther. (2017) 173:118–34. 10.1016/j.pharmthera.2017.02.011 28188812

[14] Plass C Pfister SM Lindroth AM Bogatyrova O Claus R Lichter P. Mutations in regulators of the epigenome and their connections to global chromatin patterns in cancer. Nat Rev Genet. (2013) 14:765–80. 10.1038/nrg3554 24105274

[15] Füllgrabe J Kavanagh E Joseph B. Histone onco-modifications. Oncogene. (2011) 30:3391–403. 10.1038/onc.2011.121 21516126

[16] Ma F Zhang C-y. Histone modifying enzymes: novel disease biomarkers and assay development. Expert Rev Mol Diagn. (2016) 16:297–306. 10.1586/14737159.2016.1135057 26750583

[17] Martín-Subero JI Esteller M. Profiling epigenetic alterations in disease. Adv Exp Med Biol. (2011) 711:162–77. 10.1007/978-1-4419-8216-2_12 21627049

[18] Petric R Gazic B Goricar K Dolzan V Dzodic R Besic N. Expression of miRNA and occurrence of distant metastases in patients with hurthle cell carcinoma. Int J Endocrinol. (2016) 2016:8945247. 10.1155/2016/8945247 27547222 PMC4980509

[19] Vriens MR Weng J Suh I Huynh N Guerrero MA Shen WT. MicroRNA expression profiling is a potential diagnostic tool for thyroid cancer. Cancer. (2012) 118:3426–32. 10.1002/cncr.26587 22006248 PMC6959539

[20] Dettmer MS Perren A Moch H Komminoth P Nikiforov YE Nikiforova MN. MicroRNA profile of poorly differentiated thyroid carcinomas: new diagnostic and prognostic insights. J Mol Endocrinol. (2014) 52:181–9. 10.1530/JME-13-0266 24443580 PMC4010646

[21] Sondermann A Andreghetto FM Moulatlet AC da Silva Victor E de Castro MG Nunes FD. MiR-9 and miR-21 as prognostic biomarkers for recurrence in papillary thyroid cancer. Clin Exp Metastasis. (2015) 32:521–30. 10.1007/s10585-015-9724-3 26007293

[22] de la Chapelle A Jazdzewski K. MicroRNAs in thyroid cancer. J Clin Endocrinol Metab. (2011) 96:3326–36. 10.1210/jc.2011-1004 21865360 PMC3410255

[23] Yip L Kelly L Shuai Y Armstrong MJ Nikiforov YE Carty SE. MicroRNA signature distinguishes the degree of aggressiveness of papillary thyroid carcinoma. Ann Surg Oncol. (2011) 18:2035–41. 10.1245/s10434-011-1733-0 21537871 PMC4157306

[24] Nikiforova MN Tseng GC Steward D Diorio D Nikiforov YE. MicroRNA expression profiling of thyroid tumors: biological significance and diagnostic utility. J Clin Endocrinol Metab. (2008) 93:1600–8. 10.1210/jc.2007-2696 18270258 PMC2386678

[25] Kasinski AL Slack FJ. MicroRNAs en route to the clinic: progress in validating and targeting microRNAs for cancer therapy. Nat Rev Cancer. (2011) 11:849–64. 10.1038/nrc3166 22113163 PMC4314215

[26] Galasso M Sandhu SK Volinia S. MicroRNA expression signatures in solid malignancies. Cancer J. (2012) 18:238–43. 10.1097/PPO.0b013e318258b5f4 22647360

[27] Canberk S Ferreira JC Pereira L Batista R Vieira AF Soares P. Analyzing the role of DICER1 germline variations in papillary thyroid carcinoma. Eur Thyroid J. (2020) 9:296–303. 10.1159/000509183 33718253 PMC7923931

[28] Jazdzewski K Liyanarachchi S Swierniak M Pachucki J Ringel MD Jarzab B. Polymorphic mature microRNAs from passenger strand of pre-miR-146a contribute to thyroid cancer. Proc Natl Acad Sci U S A. (2009) 106:1502–5. 10.1073/pnas.0812591106 19164563 PMC2635764

[29] Keelawat S Thorner PS Shuangshoti S Bychkov A Kitkumthorn N Rattanatanyong P. Detection of global hypermethylation in well-differentiated thyroid neoplasms by immunohistochemical (5-methylcytidine) analysis. J Endocrinol Invest. (2015) 38:725–32. 10.1007/s40618-015-0246-2 25740063

[30] Stephen JK Chen KM Merritt J Chitale D Divine G Worsham MJ. Methylation markers for early detection and differentiation of follicular thyroid cancer subtypes. Cancer Clin Oncol. (2015) 4:1–12. 10.5539/cco.v4n2p1 27158284 PMC4859763

[31] Mazeh H Mizrahi I Halle D Ilyayev N Stojadinovic A Trink B. Development of a microRNA-based molecular assay for the detection of papillary thyroid carcinoma in aspiration biopsy samples. Thyroid. (2011) 21:111–8. 10.1089/thy.2010.0356 21275764

[32] Benjamin H Schnitzer-Perlman T Shtabsky A VandenBussche CJ Ali SZ Kolar Z. Analytical validity of a microRNA-based assay for diagnosing indeterminate thyroid FNA smears from routinely prepared cytology slides. Cancer Cytopathol. (2016) 124:711–21. 10.1002/cncy.21731 27223344 PMC5096036

[33] Young NA Win KK Pomo L Anastasopoulou C Minimo C Mayrin J. An academic community hospital experience using commercially available molecular testing in the management of indeterminate thyroid nodules. J Am Soc Cytopathol. (2018) 7:92–8. 10.1016/j.jasc.2017.09.001 31043258

[34] Santos MTD Buzolin AL Gama RR Silva E Dufloth RM Figueiredo DLA. Molecular classification of thyroid nodules with indeterminate cytology: development and validation of a highly sensitive and specific new miRNA-based classifier test using fine-needle aspiration smear slides. Thyroid. (2018) 28:1618–26. 10.1089/thy.2018.0254 30319072 PMC6308280

[35] Patel KN Angell TE Babiarz J Barth NM Blevins T Duh QY. Performance of a genomic sequencing classifier for the preoperative diagnosis of cytologically indeterminate thyroid nodules. JAMA Surg. (2018) 153:817–24. 10.1001/jamasurg.2018.1153 29799911 PMC6583881

[36] Nikiforova MN Mercurio S Wald AI Barbi de Moura M Callenberg K Santana-Santos L. Analytical performance of the ThyroSeq v3 genomic classifier for cancer diagnosis in thyroid nodules. Cancer. (2018) 124:1682–90. 10.1002/cncr.31245 29345728 PMC5891361

[37] Possieri C Locantore P Salis C Bacci L Aiello A Fadda G. Combined molecular and mathematical analysis of long noncoding RNAs expression in fine needle aspiration biopsies as novel tool for early diagnosis of thyroid cancer. Endocrine. (2020). 10.1007/s12020-020-02508-w 33030666 PMC8159833

[38] American Thyroid Association Guidelines Taskforce on Thyroid N, Differentiated Thyroid C Cooper DS Doherty GM Haugen BR Kloos RT. Revised American Thyroid Association management guidelines for patients with thyroid nodules and differentiated thyroid cancer. Thyroid. (2009) 19:1167–214. 10.1089/thy.2009.0110 19860577

[39] Chou CK Chen RF Chou FF Chang HW Chen YJ Lee YF. miR-146b is highly expressed in adult papillary thyroid carcinomas with high risk features including extrathyroidal invasion and the BRAF(V600E) mutation. Thyroid. (2010) 20:489–94. 10.1089/thy.2009.0027 20406109

[40] Kunstman JW Korah R Healy JM Prasad M Carling T. Quantitative assessment of RASSF1A methylation as a putative molecular marker in papillary thyroid carcinoma. Surgery. (2013) 154:1255–61. 10.1016/j.surg.2013.06.025 24383114

[41] Niu H Yang J Yang K Huang Y. The relationship between RASSF1A promoter methylation and thyroid carcinoma: a meta-analysis of 14 articles and a bioinformatics of 2 databases (PRISMA). Medicine (Baltimore). (2017) 96:e8630. 10.1097/MD.0000000000008630 29145283 PMC5704828

[42] Mancikova V Buj R Castelblanco E Inglada-Perez L Diez A de Cubas AA. DNA methylation profiling of well-differentiated thyroid cancer uncovers markers of recurrence free survival. Int J Cancer. (2014) 135:598–610. 10.1002/ijc.28703 24382797

[43] Buj R Mallona I Diez-Villanueva A Zafon C Mate JL Roca M. Kallikreins stepwise scoring reveals three subtypes of papillary thyroid cancer with prognostic implications. Thyroid. (2018) 28:601–12. 10.1089/thy.2017.0501 29635968

[44] Klein Hesselink EN Zafon C Villalmanzo N Iglesias C van Hemel BM Klein Hesselink MS. Increased global DNA hypomethylation in distant metastatic and dedifferentiated thyroid cancer. J Clin Endocrinol Metab. (2018) 103:397–406. 10.1210/jc.2017-01613 29165662

[45] Russo D Damante G Puxeddu E Durante C Filetti S. Epigenetics of thyroid cancer and novel therapeutic targets. J Mol Endocrinol. (2011) 46:R73–81. 10.1530/JME-10-0150 21325372

[46] Xing M Usadel H Cohen Y Tokumaru Y Guo Z Westra WB. Methylation of the thyroid-stimulating hormone receptor gene in epithelial thyroid tumors: a marker of malignancy and a cause of gene silencing. Cancer Res. (2003) 63:2316–21. 12727856

[47] Hoque MO Rosenbaum E Westra WH Xing M Ladenson P Zeiger MA. Quantitative assessment of promoter methylation profiles in thyroid neoplasms. J Clin Endocrinol Metab. (2005) 90:4011–8. 10.1210/jc.2005-0313 15840741

[48] Venkataraman GM Yatin M Marcinek R Ain KB. Restoration of iodide uptake in dedifferentiated thyroid carcinoma: relationship to human Na+/I– symporter gene methylation status1. J Clin Endocrinol Metab. (1999) 84:2449–57. 10.1210/jcem.84.7.5815 10404820

[49] Neumann S Schuchardt K Reske A Reske A Emmrich P Paschke R. Lack of correlation for sodium iodide symporter mRNA and protein expression and analysis of sodium iodide symporter promoter methylation in benign cold thyroid nodules. Thyroid. (2004) 14:99–111. 10.1089/105072504322880337 15068624

[50] Xing M Tokumaru Y Wu G Westra WB Ladenson PW Sidransky D. Hypermethylation of the Pendred syndrome gene SLC26A4 is an early event in thyroid tumorigenesis. Cancer Res. (2003) 63:2312–5. 12727855

[51] Kondo T Nakazawa T Ma D Niu D Mochizuki K Kawasaki T. Epigenetic silencing of TTF-1/NKX2-1 through DNA hypermethylation and histone H3 modulation in thyroid carcinomas. Lab Invest. (2009) 89:791–9. 10.1038/labinvest.2009.50 19506552

[52] Tuncel M Aydin D Yaman E Tazebay UH Güç D Dogan AL. The comparative effects of gene modulators on thyroid-specific genes and radioiodine uptake. Cancer Biother Radiopharm. (2007) 22:281–8. 10.1089/cbr.2006.319 17600477

[53] Kitazono M Goldsmith ME Aikou T Bates S Fojo T. Enhanced adenovirus transgene expression in malignant cells treated with the histone deacetylase inhibitor FR901228. Cancer Res. (2001) 61:6328–30. 11522619

[54] Zarnegar R Brunaud L Kanauchi H Wong M Fung M Ginzinger D. Increasing the effectiveness of radioactive iodine therapy in the treatment of thyroid cancer using Trichostatin A, a histone deacetylase inhibitor. Surgery. (2002) 132:984–90. 10.1067/msy.2002.128690 12490845

[55] Fortunati N Catalano MG Arena K Brignardello E Piovesan A Boccuzzi G. Valproic acid induces the expression of the Na+/I- symporter and iodine uptake in poorly differentiated thyroid cancer cells. J Clin Endocrinol Metab. (2004) 89:1006–9. 10.1210/jc.2003-031407 14764827

[56] Furuya F Shimura H Suzuki H Taki K Ohta K Haraguchi K. Histone deacetylase inhibitors restore radioiodide uptake and retention in poorly differentiated and anaplastic thyroid cancer cells by expression of the sodium/iodide symporter thyroperoxidase and thyroglobulin. Endocrinology. (2004) 145:2865–75. 10.1210/en.2003-1258 14976143

[57] Puppin C D'Aurizio F D'Elia AV Cesaratto L Tell G Russo D. Effects of histone acetylation on sodium iodide symporter promoter and expression of thyroid-specific transcription factors. Endocrinology. (2005) 146:3967–74. 10.1210/en.2005-0128 15919754

[58] Shen WT Wong TS Chung W-Y Wong MG Kebebew E Duh Q-Y. Valproic acid inhibits growth, induces apoptosis, and modulates apoptosis-regulatory and differentiation gene expression in human thyroid cancer cells. Surgery. (2005) 138:979–85. 10.1016/j.surg.2005.09.019 16360381

[59] Hou P Bojdani E Xing M. Induction of thyroid gene expression and radioiodine uptake in thyroid cancer cells by targeting major signaling pathways. J Clin Endocrinol Metab. (2010) 95:820–8. 10.1210/jc.2009-1888 20008023 PMC2840852

[60] Shen WT Chung WY. Treatment of thyroid cancer with histone deacetylase inhibitors and peroxisome proliferator-activated receptor-gamma agonists. Thyroid. (2005) 15:594–9. 10.1089/thy.2005.15.594 16029127

[61] Delaney C Garg SK Yung R. Analysis of DNA methylation by pyrosequencing. Methods Mol Biol. (2015) 1343:249–64. 10.1007/978-1-4939-2963-4_19 26420722 PMC4772880

[62] Kristensen LS Wojdacz TK Thestrup BB Wiuf C Hager H Hansen LL. Quality assessment of DNA derived from up to 30 years old formalin fixed paraffin embedded (FFPE) tissue for PCR-based methylation analysis using SMART-MSP and MS-HRM. BMC Cancer. (2009) 9:453. 10.1186/1471-2407-9-453 20025721 PMC2804714

[63] Beikircher G Pulverer W Hofner M Noehammer C Weinhaeusel A. Multiplexed and sensitive DNA methylation testing using methylation-sensitive restriction enzymes “MSRE-qPCR”. Methods Mol Biol. (2018) 1708:407–24. 10.1007/978-1-4939-7481-8_21 29224156

[64] Zummeren MV Kremer WW Leeman A Bleeker MCG Jenkins D Sandt MV. HPV E4 expression and DNA hypermethylation of CADM1, MAL, and miR124-2 genes in cervical cancer and precursor lesions. Mod Pathol. (2018) 31:1842–50. 10.1038/s41379-018-0101-z 30135508

[65] Mitsui Y Chang I Kato T Hashimoto Y Yamamura S Fukuhara S. Functional role and tobacco smoking effects on methylation of CYP1A1 gene in prostate cancer. Oncotarget. (2016) 7:49107–21. 10.18632/oncotarget.9470 27203547 PMC5226494

[66] Spencer DH Sehn JK Abel HJ Watson MA Pfeifer JD Duncavage EJ. Comparison of clinical targeted next-generation sequence data from formalin-fixed and fresh-frozen tissue specimens. J Mol Diagn. (2013) 15:623–33. 10.1016/j.jmoldx.2013.05.004 23810758 PMC4912568

[67] Dietel M Johrens K Laffert MV Hummel M Blaker H Pfitzner BM. A 2015 update on predictive molecular pathology and its role in targeted cancer therapy: a review focussing on clinical relevance. Cancer Gene Ther. (2015) 22:417–30. 10.1038/cgt.2015.39 26358176

[68] Arreaza G Qiu P Pang L Albright A Hong LZ Marton MJ. Pre-analytical considerations for successful Next-Generation Sequencing (NGS): challenges and opportunities for Formalin-Fixed and Paraffin-Embedded Tumor Tissue (FFPE) samples. Int J Mol Sci. (2016) 17:1579. 10.3390/ijms17091579 27657050 PMC5037844

[69] Verlaat W Snijders PJF Novianti PW Wilting SM De Strooper LMA Trooskens G. Genome-wide DNA methylation profiling reveals methylation markers associated with 3q gain for detection of cervical precancer and cancer. Clin Cancer Res. (2017) 23:3813–22. 10.1158/1078-0432.CCR-16-2641 28119363

[70] Smith OK Kim R Fu H Martin MM Lin CM Utani K. Distinct epigenetic features of differentiation-regulated replication origins. Epigenet Chromatin. (2016) 9:18. 10.1186/s13072-016-0067-3 27168766 PMC4862150

[71] Bacolod MD Barany F Pilones K Fisher PB de Castro RJ. Pathways- and epigenetic-based assessment of relative immune infiltration in various types of solid tumors. Adv Cancer Res. (2019) 142:107–43. 10.1016/bs.acr.2019.01.003 30885360

[72] Wells J Farnham PJ. Characterizing transcription factor binding sites using formaldehyde crosslinking and immunoprecipitation. Methods. (2002) 26:48–56. 10.1016/S1046-2023(02)00007-5 12054904

[73] Verhelst S De Clerck L Willems S Van Puyvelde B Daled S Deforce D. Comprehensive histone epigenetics: a mass spectrometry based screening assay to measure epigenetic toxicity. MethodsX. (2020) 7:101055. 10.1016/j.mex.2020.101055 32995308 PMC7508989

[74] Chen C Ridzon DA Broomer AJ Zhou Z Lee DH Nguyen JT. Real-time quantification of microRNAs by stem-loop RT-PCR. Nucleic Acids Res. (2005) 33:e179. 10.1093/nar/gni178 16314309 PMC1292995

[75] Shi R Chiang VL. Facile means for quantifying microRNA expression by real-time PCR. Biotechniques. (2005) 39:519–25. 10.2144/000112010 16235564

[76] Liu CG Calin GA Meloon B Gamliel N Sevignani C Ferracin M. An oligonucleotide microchip for genome-wide microRNA profiling in human and mouse tissues. Proc Natl Acad Sci U S A. (2004) 101:9740–4. 10.1073/pnas.0403293101 15210942 PMC470744

[77] Meng W McElroy JP Volinia S Palatini J Warner S Ayers LW. Comparison of MicroRNA deep sequencing of matched formalin-fixed paraffin-embedded and fresh frozen cancer tissues. PLoS ONE. (2013) 8:e64393. 10.1371/journal.pone.0064393 23696889 PMC3655971

[78] Weng L Wu X Gao H Mu B Li X Wang JH. MicroRNA profiling of clear cell renal cell carcinoma by whole-genome small RNA deep sequencing of paired frozen and formalin-fixed, paraffin-embedded tissue specimens. J Pathol. (2010) 222:41–51. 10.1002/path.2736 20593407

[79] Morozova O Marra MA. Applications of next-generation sequencing technologies in functional genomics. Genomics. (2008) 92:255–64. 10.1016/j.ygeno.2008.07.001 18703132

[80] Yim JH Choi AH Li AX Qin H Chang S Tong S-WT. Identification of tissue-specific DNA methylation signatures for thyroid nodule diagnostics. Clin Cancer Res. (2019) 25:544–51. 10.1158/1078-0432.CCR-18-0841 30093451 PMC6335179

